# High levels of cell-free DNA accurately predict late acute kidney injury in patients after cardiac surgery

**DOI:** 10.1371/journal.pone.0218548

**Published:** 2019-06-18

**Authors:** Julia Merkle, Aldo Daka, Antje C. Deppe, Thorsten Wahlers, Adnana Paunel-Görgülü

**Affiliations:** Department of Cardiothoracic Surgery, Heart Center of the University of Cologne, Cologne, Germany; Policlinico S. Orsola-Malpighi, ITALY

## Abstract

Use of cardiopulmonary bypass in cardiac surgery triggers systemic inflammation by neutrophil activation leading to neutrophil extracellular traps (NETs) release. Hence, nuclear DNA released by necrotic and apoptotic cells might contribute to an increase in circulating cell-free DNA (cfDNA). cfDNA/NETs might induce endothelial damage and organ dysfunction. This study focuses on the accuracy of cfDNA to predict acute kidney injury (AKI) after on-pump surgery. 58 cardiac patients undergoing on-pump surgery were prospectively enrolled. Blood samples were taken preoperatively, immediately after surgery, at day 1, 2, 3 and 5 from patients with (n = 21) or without (n = 37) postoperative AKI development. Levels of cfDNA, neutrophil gelatinase-associated lipocalin (NGAL) and creatinine in patients’ plasma were quantified. ROC curves were used to assess the predictive value of the biomarkers for AKI. Further baseline characteristics and perioperative variables were analyzed.cfDNA and NGAL levels highly increased in AKI patients and significant intergroup differences (vs. non-AKI) were found until day 3 and day 5 after surgery, respectively. cfDNA levels were significantly elevated in patients who developed late AKI (>24 hours), but not in those with AKI development during the first 24 hours (early AKI). NGAL and creatinine, which were highest in patients with early AKI, accurately predicted during the first 24 postoperative hours (early AKI). At day 3, at a threshold of 260.53 ng/ml cfDNA was the best predictor for AKI (AUC = 0.804) compared to NGAL (AUC = 0.699) and creatinine (AUC = 0.688). NGAL, but not cfDNA, was strongly associated with AKI stages and mortality. Monitoring of cfDNA levels from the first postoperative day might represent a valuable tool to predict late AKI after on-pump surgery.

## Introduction

Acute kidney injury (AKI) is widely recognized as a common complication of cardiac surgery and is associated with morbidity and mortality [1]. The use of cardiopulmonary bypass (CPB) during cardiac surgery and activation of blood cells by the non-physiological surfaces of filters and elements of the extracorporeal circuit initiates a systemic inflammatory response (SIRS), contributing to the development of postsurgical complications including AKI. Indeed, a long operation time and CPB duration have been reported as risk factors for AKI [2–4]. According to the definition and surgery type, the incidence of postoperative AKI varies from 5 to 42% [1, 5]. As there is no effective therapy available for AKI after cardiac surgery, identifying novel and sensitive biomarkers able to detect AKI prior to its clinical diagnosis would be beneficial and might allow development of new preventive strategies. Most frequently studied and promising markers are plasma neutrophil gelatinase-associated lipocalin (NGAL) [6, 7] and IL-18 [8].

Recently, circulating cell-free DNA (cfDNA) has received increasing attention as a danger-associated molecular pattern and has been used as a clinical marker in cancer [9], trauma [10] and sepsis [11, 12] among others. cfDNA becomes released from necrotic and apoptotic cells as well as by activated neutrophils by a process called NETosis. Upon activation by proinflammatory cytokines, activated platelets [13], activated endothelial cells [14] or microorganisms, neutrophils release extracellular chromatin fibers in the form of neutrophil extracellular traps (NETs) [15], which are decorated with neutrophil granular proteins such as elastase and myeloperoxidase and trap invading microorganisms. However, excessive NETs release in the circulation may result in tissue injury and has been linked to cytotoxicity [16], thrombosis [17] and autoimmunity [18]. Previous studies have demonstrated that circulating cfDNA concentrations reflect the amount of NETs release in the blood [19, 20]. Hence, in multiple trauma patients, the concentration of circulating cfDNA/NETs correlates with the severity of injury [19]. Recently, serum cfDNA/NETs was found to be markedly elevated after cardiac surgery and correlation with perioperative renal dysfunction was reported [21]. Further on, a meta-analysis reported that leukocyte filter application reduced renal injury in cardiac surgery patients [22], supporting the hypothesis that neutrophils and plasma cfDNA/NETs might play a role for the pathogenesis and development of AKI. There is a strong need to identify suitable early and reliable markers for prediction of the development of postoperative kidney dysfunction. However, suitability and feasibility of plasma cfDNA/NETs as a marker for AKI development after cardiac surgery are currently not clear. In the present study, we analyzed perioperative plasma levels of cfDNA in patients undergoing cardiac surgery with CPB and subsequent AKI development and evaluated the feasibility of cfDNA as a predictor of kidney injury. We further compared the predictive value of cfDNA with the well-established AKI biomarkers plasma NGAL and creatinine.

## Materials and methods

This study was approved by the Ethics Committee of the Medical Faculty of the University of Cologne (#17–205). Written informed consent was obtained from all participants at the time of admission. Fifty-eight patients undergoing on-pump cardiac surgery with estimated CPB duration ≥80 min and aged 60 years or older were enrolled prospectively in this study from September 2017 to April 2018. Patients were followed up until August 2018. Exclusion criteria for the study were: age <60 years, patients undergoing hemodialysis; patients with immunological disorders or systemic immunosuppression, infection, cancer, pregnancy or patients who refused study participation.

AKI was defined according to the AKIN (acute kidney injury network) classification system [23] within the first five days post-surgery. Stage 1 AKI was an increase of blood creatinine ≥0.3 mg/dL within 48 hours or at least 50% increase from baseline. Stage 2 was a 2-fold increase from baseline creatinine. Stage 3 AKI is defined as a 3-fold increase from baseline creatinine or increase to ≥4.0 mg/dL.

The preoperative risk for operative mortality was evaluated by means of the additive and logistic EuroSCORE II.

Blood samples were collected at the day of admission, immediately after surgery and postoperatively at day 1 (22–24 hours post-surgery), day 2 (46–48 hours post-surgery), day 3 (70–72 hours post-surgery) and day 5 (118–120 hours post-surgery). Plasma samples were harvested by centrifugation (10 min at 3000 × g) and stored at -80°C until further processing.

### Quantification of plasma cfDNA

Levels of cfDNA were quantified by Pico green staining as previously described [20].

### Quantification of NGAL by ELISA

Plasma neutrophil gelatinase-associated lipocalin (NGAL) levels were determined by using the Human Lipocalin-2/NGAL Duo Set Elisa (R&D Systems, Wiesbaden-Nordenstadt, Germany) according to the manufacturer’s instructions.

### Statistical analyses

Statistical analyses were performed using GraphPad Prism 5 software (Graphpad Software Inc., San Diego, CA, USA), MedCalc software and IBM SPSS Statistics for Windows, Version 25 (IBM Corp. Released 2017. Armonk, NY: IBM Corp). Patient data are presented as box plots representing median (heavy line in boxes) with 25^th^ and 75^th^ percentiles. Whiskers indicate the minimum and maximum values, respectively. Statistical comparisons of continuous variables were performed using non-parametric Mann-Whitney U test for non-normally distributed variables, whereas categorical variables were assessed using Pearson’s χ² test or Fisher’s exact test depending on the minimal expected count in each crosstab. Differences between more than two groups of non-parametric distributed data were determined by the Friedman test with Dunn as post hoc test. Correlations were evaluated by the Spearman correlation coefficient (*r*).

Areas under the curve (AUC) of receiver operating characteristic (ROC) curves were determined and optimal cut-off values for plasma cfDNA, NGAL and creatinine levels for predicting AKIs were evaluated. The optimal cfDNA, NGAL and creatinine cut-off values were defined as the value that provided the highest sensitivity and specificity for predicting AKI. Univariate logistic regression was used to evaluate independent predictors of AKI after cardiac surgery. *P* ≤ 0.05 was considered statistically significant.

## Results

### Characteristics of the study cohort

Among all patients included in this study, 21 patients developed AKI (36%) after cardiac surgery, whereas 37 had an uneventful outcome. Baseline demographics and clinical variables are summarized in Table 1. There were no statistically significant differences in terms of baseline characteristics. Seven patients (33%) developed AKI during the first 24 hours after surgery (defined as early AKI), four patients (19%) at day 2, two patients (10%) at day 3 and eight patients (38%) at day 5 after surgery. In the AKI group, 14 patients (67%) suffered from mild postoperative AKI (stage 1) and 7 patients (33%) had moderate AKI (stage 2). No patient developed stage 3 AKI. Four patients with stage 2 AKI showed creatinine rinse at day 1 after surgery and three patients were diagnosed with stage 2 AKI at day 2. Four patients with stage 2 AKI and one patient with stage 1 AKI died postoperatively. Of these patients, three patients developed stroke finally leading to death. One patient suffered from reduced left ventricular ejection fraction of 33% post-surgery provoking kidney injury and subsequent multiple organ failure. Furthermore, one patient underwent re-exploration due to excessive postoperative bleeding and died due to development of AKI and multiple organ failure.

**Table 1:** Association of demographics and preoperative situation for non-AKI group an AKI group.

	non AKI group n = 37	AKI group n = 21	p-value
Age	70.0 (65.5;76.0)	72.0 (69.;78)	0.156
Height (cm)	171.0 (163;175)	170.0 (167;180)	0.422
Weight (kg)	77.0 (68;84)	79.0 (71;99)	0.190
Body mass index (kg/m²)	26.5 (24;30)	27.5 (24;32)	0.419
Female gender n (%)	13 (35.1)	5 (23.8)	0.370
Male gender n (%)	24 (64.9)	16 (76.2)	0.370
LVEF >55% n (%)	30 (81.1)	15 (71.4)	0.515
Euroscore	6.0 (4;7)	7.0 (5;9)	0.086
Smoker/former Smoker n (%)	20 (54.1)	10 (47.6)	0.637
Hyperlipidemia n (%)	31 (83.8)	20 (95.2)	0.403
Triple vessel desease n (%)	18 (48.6)	8 (38.1)	0.437
Left main coronary artery stenosis n (%)	10 (27.0)	8 (38.1)	0.381
Carotid artery stenosis >50% n (%)	3 (8.1)	2 (9.5)	1.000
Prior hypertension n (%)	36 (97.3)	21 (100)	1.000
Prior myocardial infarction n (%)	8 (21.6)	3 (14.3)	0.729
Prior stroke n(%)	0 (0)	1 (4.8)	0.362
Prior cardiac surgery n (%)	2 (5.4)	5 (23.8)	0.086
Diabetes mellitus n (%)	11 (29.7)	7 (33.3)	0.776
COPD n (%)	4 (10.8)	4 (19.0)	0.443
Peripheral artery desease n (%)	6 (16.2)	6 (28.6)	0.320
Pulmonary hypertension n (%)	5 (13.5)	4 (19.0)	0.710
Preoperative beta blocker n (%)	20 (54.1)	15 (71.4)	0.194
Preoperative ACE inhibitor n (%)	15 (40.5)	9 (42.9)	0.863
Preoperative aspirin n (%)	28 (75.7)	15 (71.4)	0.723
Preoperative diuretics n (%)	12 (32.4)	11 (52.4)	0.136
Preoperative statin n (%)	26 (70.3)	18 (85.7)	0.187
Preoperative insulin n (%)	3 (8.1)	5 (23.8)	0.124
Preoperative antidiabetics n (%)	9 (24.3)	6 (28.6)	0.723

AKI, acute kidney injury; COPD, chronic obstructive pulmonary disease; LVEF, left ventricular ejection fraction.

Table 2 gives an overview about the cardiac surgical procedures. Patients in the AKI group had significantly prolonged operation, bypass and aortic cross clamp time. Hence, the number of patients receiving venous grafts was significantly lower. Patients included in the AKI group were mechanically ventilated for a prolonged period, had a longer stay on the ICU and a higher mortality rate. Three patients required renal replacement therapy.

**Table 2:** Association of operative strategy for non-AKI group and AKI group.

	non AKI group	AKI group	p-value
CABG n(%)	15 (71.4)	6 (28.6)	0.362
AV replacement n(%)	2 (5.4)	1 (4.8)	1.000
CABG+AV repair	1 (2.7)	0 (0.0)	1.000
CABG+AV replacement	9 (24.3)	2 (9.5)	0.296
MV repair	2 (5.4)	0 (0.0)	0.530
MV replacement+TV repair	2 (5.4)	2 (9.5)	0.615
MV repair+TV replacement	0 (0.0)	1 (4.8)	0.362
MV repair+AV replacement	1 (2.7)	0 (0.0)	1.000
CABG+MV replacement	0 (0.0)	2 (9.5)	0.127
Aortic surgery+AV replacement	3 (8.1)	3 (14.3)	0.657
AV replacement +MV replacement	0 (0.0)	1 (4.8)	0.362
Aortic surgery +CABG	1 (2.7)	2 (9.5)	0.547
Duration of operation (h)	3.5 (3;4)	4.5 (4;6.0)	**<0.001**
Bypass time (min)	100.0 (82;115)	147.0 (101;209)	**0.001**
Aortic cross clap time (min)	62.0 (46;80)	96.0 (69;126)	**0.006**
Number of anastomoses	2.0 (0.0;3.5)	2.0 (0.0;3.5)	0.785
Venous graft	23 (65.7)	12 (34.3)	**0.003**
Ventilation time (h)	11.0 (8.0;17.5)	17.0 (14;32)	**0.004**
Intensive care unit stay (d)	1.5 (1.0;3.0)	3.0 (2.5;8.5)	**0.025**
1-year Mortality	0 (0.0)	5 (23.8)	**0.004**

AKI, acute kidney injury; AV, aortic valve; CABG, coronary artery bypass graft; MV, mitral valve; PVL, paravalvular leak; TV, tricuspid valve.

### Perioperative changes of cfDNA and NGAL levels in patients undergoing on-pump surgery

**Fig 1:**
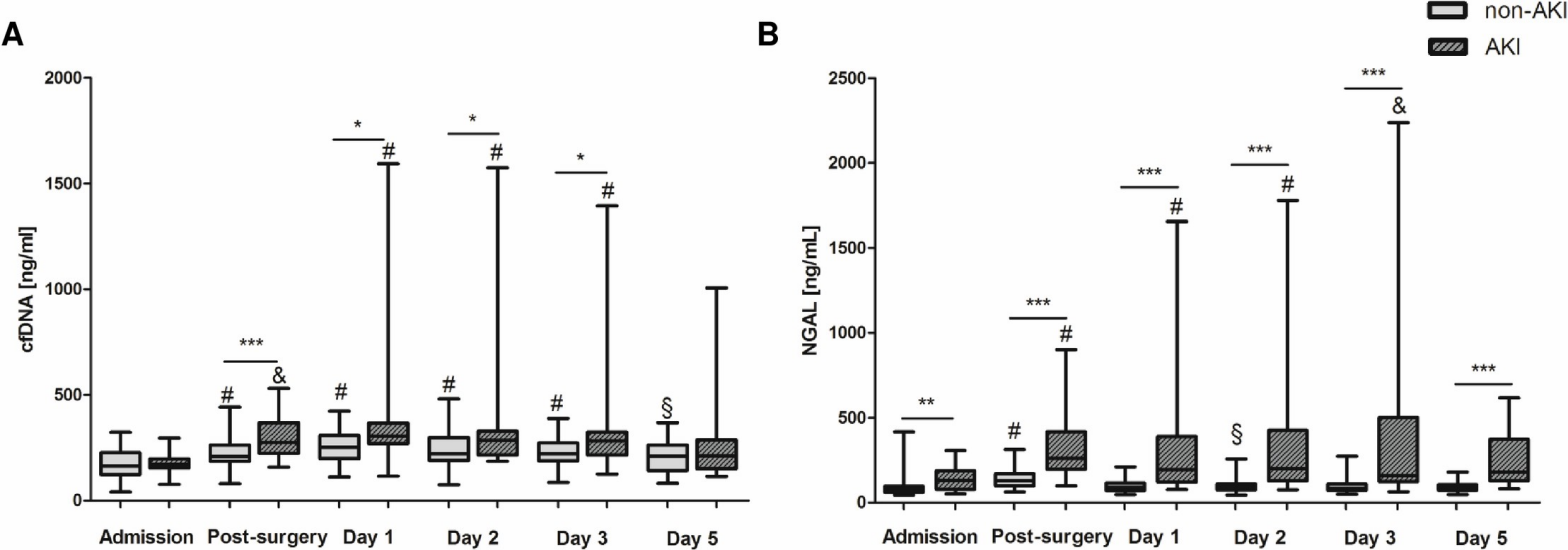
Kinetics of cfDNA/NETs and NGAL in patients undergoing cardiac surgery with CPB. cfDNA (A) and NGAL (B) levels were quantified in plasma of patients with (n = 21,) or without (n = 37) postoperative AKI development at the indicated times. &p<0.05, §p<0.01, #p<0.001 vs. admission; *p<0.05, **p<0.01, ***p<0.001.

Plasma levels of cfDNA and NGAL were quantified at the day of admission, after surgery, as well as at day 1, day 2, day 3 and day 5 after surgery. As depicted in Fig 1, cfDNA levels significantly increased after surgery in all patients, i.e. those with and without kidney injury (Fig 1A). Of note, patients who developed AKI during the first 5 days after surgery showed significantly elevated cfDNA levels immediately after surgery, at day 1, day 2 and day 3 when compared to control patients with uneventful outcome. Similarly, NGAL also significantly increased after surgery and significant intergroup differences were found at least until day 5 after surgery (Fig 1B). However, in patients with postsurgical AKI development, NGAL levels were found to be already significantly increased at the day of admission suggesting a higher predisposition for postsurgical complications.

Positive correlation between cfDNA and plasma NGAL in patients with AKI development was already found immediately after surgery (Spearman’s Rho = 0.445, p = 0.04). However, cfDNA did not correlate with NGAL at other times and no correlation with creatinine was found. Correlation of cfDNA and NGAL was not detected in patients without AKI.

### Comparison between prognostic ability of plasma cfDNA, NGAL and creatinine for AKI after cardiac surgery

**Fig 2:**
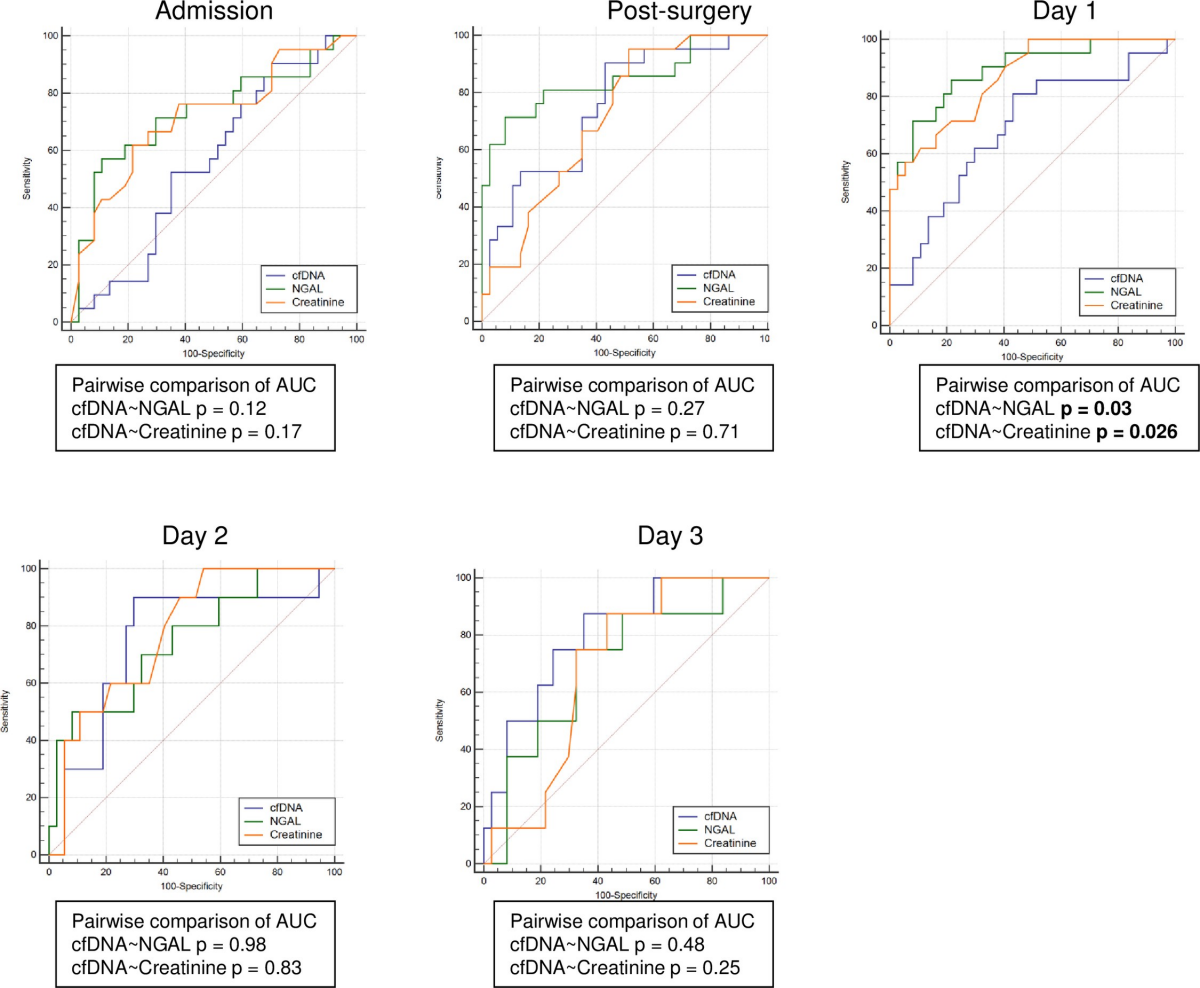
ROC curves. ROC curves at admission, post-surgery, day 1, day 2 and day 3 for AKI prediction.

To study the feasibility of cfDNA as an AKI biomarker, receiver operating characteristic (ROC) analyses were performed. We further compared the area under the ROC curve (AUC) between plasma cfDNA, NGAL and creatinine at different time points (Fig 2). Moderate rise in serum creatinine levels after cardiac surgery has already been demonstrated to be associated with postsurgical AKI [24–26]. NGAL levels were highly predictive for AKI development at admission, after surgery and at day 1 post-surgery (Table 3). At day 1, a significant reduction in the AUC for cfDNA was found when compared with the AUCs for NGAL and creatinine, respectively. However, with the exception of day 1, the AUCs did not significantly differ. Interestingly, in the further course, the AUCs for cfDNA increased reaching a maximum at day 3 after surgery. At a cut-off value of 269.34 ng/ml at day 2 and 260.53 ng/ml at day 3 highest sensitivity of 90% and 87.5% as well as specifity of 70.3% and 64.9% were determined. Diagnostic odds ratio (DOR) at day 2 and day 3 were highest for cfDNA (21.64 at day 2 and 13.1 at day 3) when compared to NGAL (11.42 at day 2 and 6.24 at day 3). Furthermore, AUCs for creatinine levels were almost continuously lower when compared to the AUCs for NGAL (Table 3).

**Table 3:** Diagnostic accuracy of cfDNA, NGAL and creatinine for predicting AKI after on-pump surgery.

	Predictor	AUC	95% CI	p-value	Cut-off	Sensitivity	Specificity	+LR	-LR	DOR
Admission	*cfDNA*	0.561	0.425–0.691	0.426	132.21	90.48	29.37	1.29	0.32	4.03
	*NGAL*	0.732	0.6–0.84	**0,0019**	116,28	57,1	89,2	5.29	0.48	11.02
	*Creatinine*	0.718	0.585–0.828	**0,003**	0,99	61,9	78,4	2.86	0.49	5.83
Post-surgery	*cfDNA*	0.754	0.623–0.858	**0,0001**	211,94	90,5	56,8	2.09	0.17	12.29
	*NGAL*	0.846	0.727–0.927	**<0,0001**	226,98	71,43	91,9	8.81	0.31	28.41
	*Creatinine*	0.719	0.585	**0,001**	0,77	95,2	48,65	1.85	0.098	18.87
Day 1	*cfDNA*	0.686	0.551–0.8	**0,013**	267,69	80,95	56,76	1.87	0.34	5.5
	*NGAL*	0.891	0.781–0.957	**<0,0001**	115,87	85,71	78,38	3.96	0.18	22
	*Creatinine*	0.866	0.75–0.94	**<0,0001**	1.09	57.14	94.59	10.57	0.45	23.48
Day 2	*cfDNA*	0.749	0.601–0.864	**0,0087**	269,34	90	70,3	3.03	0.14	21.64
	*NGAL*	0.746	0.598–0.862	**0,0075**	146,1	50	91,9	6.17	0.54	11.42
	*Creatinine*	0.776	0.63–0.884	**0,0002**	0,79	100	45,9	1.85	0	
Day 3	*cfDNA*	0.804	0.659–0.907	**0,0002**	260,53	87,5	64,9	2.49	0.19	13.1
	*NGAL*	0.699	0.544–0.827	0,056	95,79	75	67,6	2.31	0.37	6.24
	*Creatinine*	0.688	0.532–0.817	**0,027**	0,86	87,5	56,8	2.02	0.22	9.18

AKI, acute kidney injury; AUC, area under curve; cfDNA, cell-free DNA; CI, confidence interval; DOR, diagnostic odds ratio; LR, likelihood ratio; NGAL, neutrophil gelatinase-associated lipocalin.

### Association of cfDNA and NGAL with late AKI development

**Fig 3:**
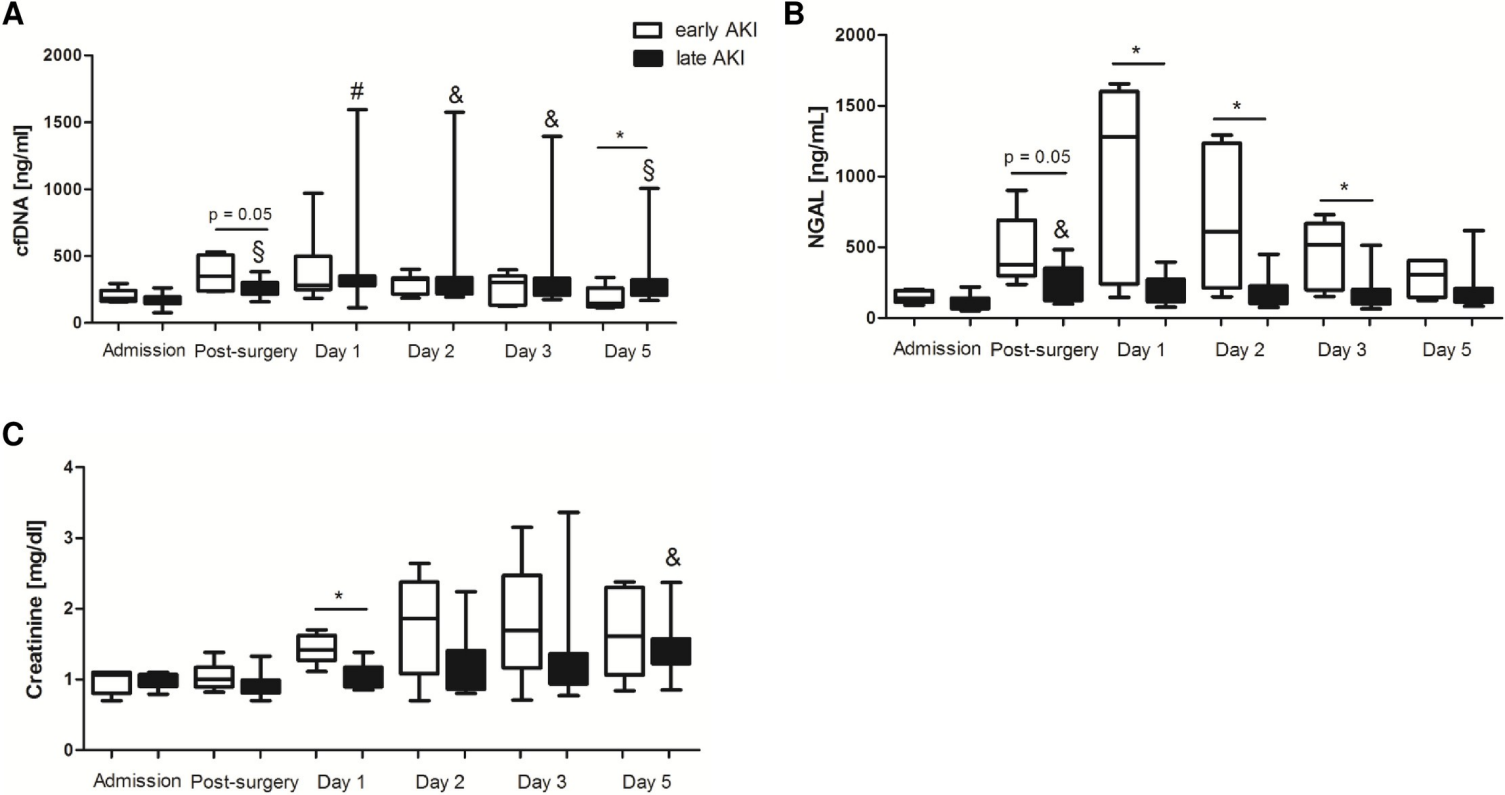
Kinetics of cfDNA/NETs, NGAL and creatinine in patients with early and late postoperative AKI development. AKI patients were divided into those with diagnosed AKI during the first 24 hours after surgery (early AKI) and those with AKI development at later times (late AKI). cfDNA (A), NGAL (B) and creatinine (C) concentrations were quantified at the defined times. &p<0.05, §p<0.01, #p<0.001 vs. admission; *p<0.05.

To prove if cfDNA might be associated with AKI at late stages after cardiac surgery, we compared cfDNA and NGAL levels between patients with early and those with late AKI development. Early postoperative AKI was defined as elevation in creatinine levels according to the AKIN criteria during the first 24 hours after surgery, whereas patients displaying increase in creatinine levels at later times were included in the late AKI group. Both, cfDNA and NGAL levels significantly increased after surgery in patients with late AKI development when compared to baseline levels (Fig 3) being in line with the significant positive correlation found between cfDNA and NGAL. At day 1, NGAL levels were significantly higher in patients included in the early AKI group when compared to the levels detected in patients with late AKI. In contrast, cfDNA was only significantly increased in patients with late AKI development. cfDNA levels remained increased until at least postoperative day 5. More importantly, only cfDNA plasma levels, but not NGAL or creatinine, were significantly elevated in AKI patients at day 5 after surgery in comparison to patients with AKI development during the first 24 hours after surgery (early AKI). cfDNA levels in patients who developed AKI at day 5 after surgery showed positive correlation with plasma NGAL (Spearman’s Rho 0.76, p = 0.03) and creatinine (Spearman’s Rho 0.82, p = 0.03). However no correlations were found in patients of the early AKI group nor in patients with late AKI development.

### Association of cfDNA and NGAL with the severity of AKI and mortality

**Fig 4:**
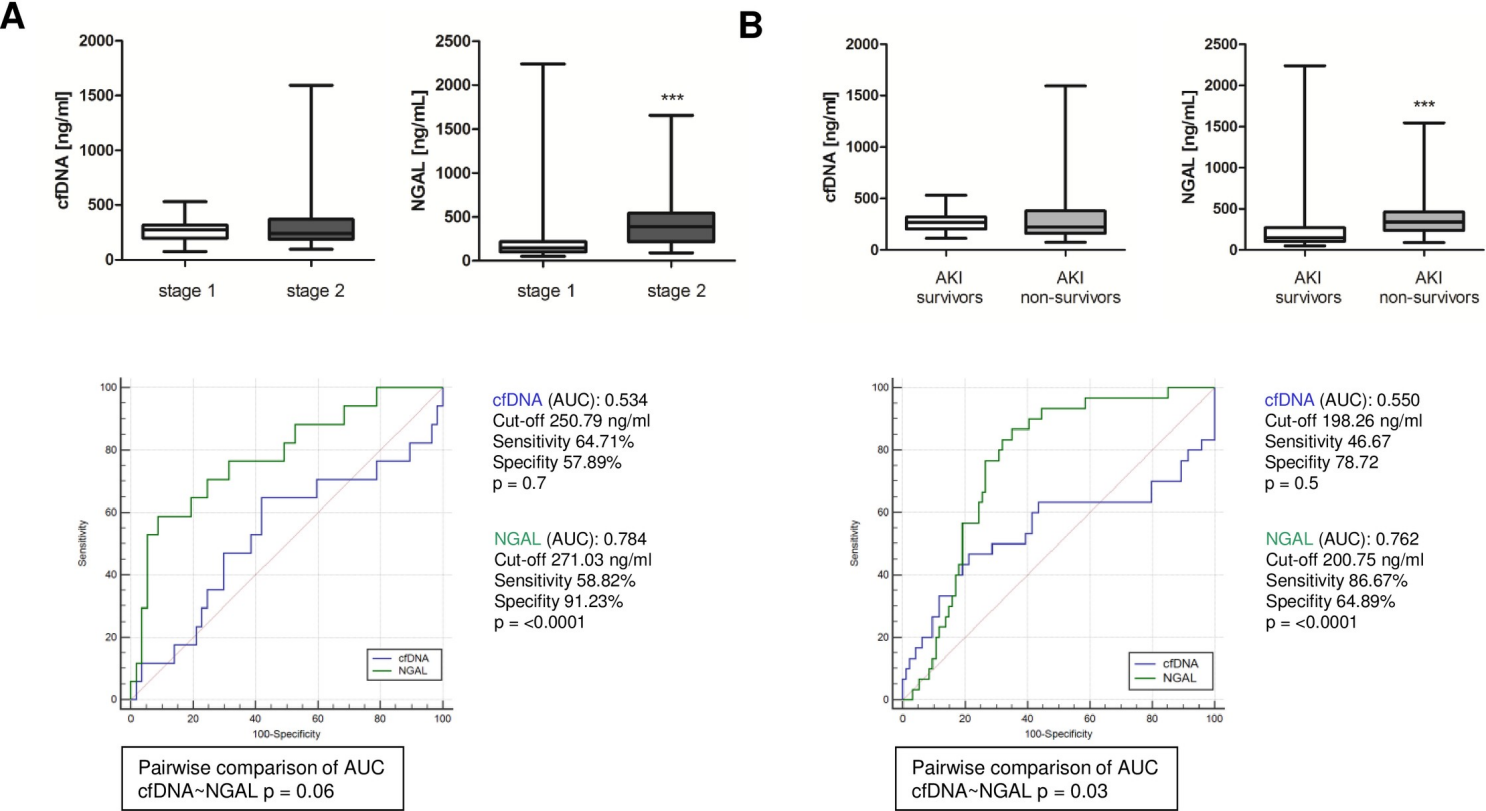
Association of disease severity and mortality with cfDNA and NGAL. (A) cfDNA and NGAL were compared between patients with stage 1 (n = 14) and stage 2(n = 7) AKI. Receiver operating characteristic curves were performed for the prediction of tsgae 2 AKI. (B) cfDNA and NGAL levels in patients in survivors and patients who died during 1-year follow-up. Receiver operating characteristic curves were performed for the prediction of mortality in AKI patients.

To further elaborate if plasma cfDNA might be associated with disease severity or mortality, respectively, we first compared all cfDNA concentrations determined in patients with stage 1 AKI with the concentrations found in stage 2 AKI patients (Fig 4A). No intergroup differences could be found. In contrast, patients who developed stage 2 AKI had significantly higher levels of circulating NGAL compared to patients with stage 1 AKI. By performing ROC curve analysis, NGAL was found to be a good predictor for stage 2 AKI (Cut-off 271.03 ng/ml, AUC = 0.784) among patients with kidney injury. Similarly NGAL, but not cfDNA, was significantly higher in patients who did not survive after cardiac surgery (Fig 4B). AUC for NGAL was 0.762 and significantly higher than the AUC for cfDNA (AUC = 0.550). Thus, high NGAL levels might indicate more severe kidney injury. Additionally, NGAL levels can be used to predict mortality of patients who develop AKI during the first 5 days after cardiac surgery.

### Univariate analysis

By performing univariate regression analysis, significant prediction for postoperative occurrence of AKI on basis of cfDNA for values greater than threshold was found for hyperlipidemia at surgery day odds ratio = 12.00 [1.13–107] (Table 4). We also found that on the basis of NGAL (> threshold value), surgery duration, bypass time, cross-clamping time and ICU stay at surgery day, day 1 and day 2 were significantly associated with AKI. At the third postoperative day, bypass and cross-clamping time, ICU stay and hyperlipidemia were independent AKI predictors (Table 5).

**Table 4:** Predictors for postoperative occurrence of AKI. Odds ratios refer to cfDNA values greater than threshold for AKI development.

Variables	cfDNA at admission	cfDNA at OP day	cfDNA at day 1	cfDNA at day 2	cfDNA at day 3
Surgery duration (h)^b^	0.85 (0.25–2.95)	1.77 (0.60–5.24)	1.48 (0.51–4.30)	0.76 (0.26–2.22)	0.94 (0.33–2.68)
Bypass time (min)^b^	1.50 (0.40–5.62)	2.39 (0.76–7.49)	1.40 (0.47–4.16)	1.09 (0.37–3.21)	1.04 (0.36–3.03)
Cross-clamping time (min)^b^	0.75 (0.22–2.59)	1.38 (0.48–3.96)	1.18 (0.42–3.37)	0.97 (0.34–2.78)	0.86 (0.30–2.42)
ICU stay (d) ^b^	1.35 (0.32–5.73)	0.79 (0.25–2.54)	0.87 (0.27–2.80)	1.80 (0.56–5.77)	0.94 (0.30–3.00)
Ventilation time (h) ^b^	0.86 (0.82–9.00)	1.59 (0.12–10.08)	2.23 (0.22–22.80)	5.10 (0.50–52.40)	0.39 (0.38–3.96)
Hyperlipidemia	6.22 (1.18–32.76)^a^	12.00 (1.13–107)^a^	4.31 (0.89–11.48)	4.55 (0.51–40.60)	1.010 (0.22–5.40)

^a^ significant predictors in univariate analysis.

^b^ value>mean. Odds Ratio with 95% confidence interval (CI); cfDNA cell-free DNA.

**Table 5:** Predictors for postoperative occurrence of AKI. Odds ratios refer to NGAL values greater than threshold for AKI development.

Variables	NGAL at admission	NGAL at OP day	NGAL at day 1	NGAL at day 2	NGAL at day 3
Surgery duration (h)^b^	4.40 (1.28–15.15) ^a^	4.88 (1.49–15.90) ^a^	3.56 (1.19–10.59) ^a^	5.73 (1.75–18.75) ^a^	2.88 (0.97–8.56)
Bypass time (min)^b^	2.87 (0.88–9.38)	10.85 (3.01–39.15) ^a^	8.84 (2.57–30.41) ^a^	8.75 (2.54–30.06) ^a^	5.34 (1.61–17.76) ^a^
Crossclamping time (min)^b^	7.80 (1.91–31.79) ^a^	7.50 (2.07–27.20) ^a^	8.21 (2.53–26.68) ^a^	8.67 (2.38–31.54) ^a^	3.17 (1.08–9.31) ^a^
ICU stay (d) ^b^	4.25 (1.22–14.78) ^a^	6.11 (1.75–21.37) ^a^	5.40 (1.48–19.73) ^a^	12.75 (3.24–50.12) ^a^	10.29 (2.07–51.22) ^a^
Ventilation time (h) ^b^	9.46 (0.91–98.97)				
Hyperlipidemia	0.95 (0.16–5.45)	3.27 (0.37–29.35)	1.19 (0.24–5.84)	0.67 (0.13–3.32)	0.84 (0.17–4.16) ^a^

^a^ significant predictors in univariate analysis.

^b^ value>mean. Odds Ratio with 95% confidence interval (CI); NGAL neutrophil gelatinase-associated lipocalin.

## Discussion

AKI after cardiac surgery is a widespread and serious postoperative complication that is closely associated with a higher risk of early and also late mortality. During the last decades, many studies have been conducted to identify new serum and urinary biomarkers of kidney injury to improve AKI diagnosis [27–29].

Use of CPB during cardiac surgery triggers release of cfDNA/NETs and significant increase of cfDNA/NETs immediately after on-pump surgery has recently been reported by our group and others [20, 30]. Similar kinetics were previously reported in patients with multiple trauma and sepsis [19] and cfDNA has been proposed as a prognostic tool for mortality in severely injured patients [31]. Use of CPB induces systemic inflammation leading to the activation of neutrophils and NETs formation culminating in endothelial and organ damage, e.g. AKI. Additionally, cfDNA might become released by damaged cells and amplify inflammation by the activation of TLR9 receptor [32, 33]. Other proinflammatory mechanisms mediated by circulating cfDNA/NETs comprise tubular cell necrosis and renal inflammation by the interaction of histones and TLR-2 and TLR-4 [34]. Here, we found cfDNA/NETs to strongly increase after cardiac surgery and, in contrast to previously published data [35], significant differences regarding plasma cfDNA concentrations between patients with and without AKI development were found until day 3 after surgery. However, cfDNA levels did not correlate with disease severity or mortality and its prognostic value for AKI was found to increase when surgery-triggered acute inflammation subsides. A very recent published report describes that ischemia/reperfusion injury induces NETs formation in the kidney yielding in higher levels of circulating cfDNA. The same group also demonstrated that NETs degradation or inhibition of NETs formation is sufficient to ameliorate kidney injury [36]. As mentioned above, these findings cannot be supported by our data. The discrepancies between the studies might be explained by the fact that in cardiac surgery, cfDNA does not seem to solely indicate kidney injury but rather is more likely to reflect the systemic inflammation. This assumption is assured by the finding that cfDNA levels significantly increased in both patients groups, e.g. in patients with and without AKI development. Rather, cfDNA levels were also found to depend on the time of CPB support, largely in part due to massive neutrophil activation by the extracorporeal circuit and subsequent NETs release [20].

In fact, by comparing cfDNA with plasma NGAL, NGAL was found to be a reliable and sensitive biomarker to predict AKI at a very early stage after surgery. In line with the study of Perrotti *et al*., NGAL levels at admission and also at day 1 after surgery accurately predicted AKI development [6], whereas at later times, i.e. >24 hours, the AUC values started to decline. However, as stage 2 AKI was diagnosed at day 1 and day 2 after surgery, respectively, NGAL levels were also highly predictive for disease severity and mortality. These findings are in line with the data presented by Bennett and co-workers who demonstrated that NGAL is an early predictive biomarker of AKI severity after CPB [37].

While changes of creatinine levels have been described to occur late in the development of AKI, there is some evidence for a rise in serum creatinine to accurately predict AKI after cardiac surgery [24]. In accordance to previous reports [25, 26, 38], demonstrating that assessment of serum creatinine has a good predictive value for cardiac surgery-associated AKI, our data suggest that serum creatinine is strongly associated with AKI development after cardiac surgery as reliably as NGAL. In fact, except for day 1, no significant differences between AUC values of cfDNA, NGAL and creatinine were found. However, use of serum creatinine in cardiac surgery with CPB should be treated with caution as creatinine levels might be unreliable. On the one hand, creatinine levels become modulated by hypothermia muscle damage during surgery. On the other hand, creatinine concentrations determined post-surgery do not really reflect the in vivo concentration and are usually lower because of patients’ volume therapy and hemodilution [39].

More interestingly, we show here for the first time, that cfDNA/NETs but not NGAL or creatinine represent valuable AKI predictors at late stage after cardiac surgery. Indeed, patients with AKI development >24 hours after surgery, displayed a more pronounced rise in cfDNA after surgery compared to patients with early AKI development during the first 24 hours, and significant intergroup differences were found at day 5. Of note, neither NGAL nor creatinine showed a similar course, and both biomarkers were found to be significantly higher in patients with early AKI. In patients with AKI development at postoperative day 5, for the day 3 cfDNA quantification, the AUC was 0.804 and the diagnostic odds ratio for AKI prediction was 13.1 using a cut-off value of 260.53 ng/ml. In turn, AUCs for NGAL and creatinine (0.699 and 0.688) as well as diagnostic odds ratios of 6.24 and 9.18 were lower.

The underlying mechanisms could not be determined by the current study design. Also the source of cfDNA in patients with late AKI was not closer identified. We assume that plasma cfDNA at early stages after surgery originates from necrotic tissue and hyperactivated neutrophils and largely reflects systemic inflammation. Conversely, when surgery-associated inflammation starts to subside, cfDNA probably originating from necrotic tubular epithelial cells, functions to a great extent as a biomarker for kidney injury. Indeed, levels for neutrophil elastase, IL-6 and TNF-α in patients undergoing cardiac surgery have been demonstrated to peek during the first hours after surgery and visibly declined until the fourth postoperative day [40]. AKI development on day 5 after surgery might rather be a consequence of postoperative patient management such as exposure to nephrotoxic drugs. In this regard, combined administration of ACE inhibitors, angiotensin receptor blockers, diuretics, or nonsteroidal anti-inflammatory drugs, as part of postoperative management, were found to be associated with increased risk of AKI [41, 42]. However, early and exact detection of AKI is critical in clinical practice and would ensure clinical interventions, such as avoidance of hypovolemia or aggressive diuresis which might contribute to the reduction of renal damage. Thus, it is likely, that timely diagnosis on the basis of early biomarkers, e.g. NGAL, could be efficient in limiting kidney injury progression and subsequent rise in cfDNA.

It should be also mentioned, that in patients with post-surgical AKI development NGAL and creatinine were already significantly increased at the time of admission, probably due to stronger inflammation in these patients, although levels of CRP were not elevated (unpublished results). Thus, it might be assumed that patients with higher inflammation or consisting signs of kidney injury, already preoperatively, are more prone for postsurgical complication, e.g. AKI. Indeed, both NGAL and creatinine expression are influenced by comorbidities [43, 44]. Additionally, despite the limited patient number included in this study, our results strongly support previously published data demonstrating increased mid-term / long-term mortality in patients with AKI after cardiac surgery [45, 46].

However, there are also some limitations of this study. First, data have been collected from a single center with a non-blinded design with a limited number of patients. Our AKI diagnosis is solely based on serum creatinine and we did not consider changes in urinary biomarkers. Previous studies identified urinary IL-18 and NGAL, among others, as early predictive markers for AKI after CPB [29, 47] Taking into account that volume overload might falsify serum creatinine, this may represent a further limitation of this study.

Further large-scale analyses will be needed to illustrate the relationship between plasma cfDNA and postoperative AKI development after on-pump surgery and to straighten its feasibility as an AKI biomarker. Nevertheless, cfDNA should be considered as a feasible biomarker to predict AKI in cardiac surgical patients.

## References

[1] Hobson CE, Yavas S, Segal MS, Schold JD, Tribble CG, Layon AJ, et al Acute kidney injury is associated with increased long-term mortality after cardiothoracic surgery. Circulation. 2009;119(18):2444–53. 10.1161/CIRCULATIONAHA.108.800011 . 19398670

[2] Bove T, Calabro MG, Landoni G, Aletti G, Marino G, Crescenzi G, et al The incidence and risk of acute renal failure after cardiac surgery. J Cardiothorac Vasc Anesth. 2004;18(4):442–5. . 15365924 10.1053/j.jvca.2004.05.021

[3] Roh GU, Lee JW, Nam SB, Lee J, Choi JR, Shim YH. Incidence and risk factors of acute kidney injury after thoracic aortic surgery for acute dissection. Ann Thorac Surg. 2012;94(3):766–71. 10.1016/j.athoracsur.2012.04.057 . 22727320

[4] Kumar AB, Suneja M, Bayman EO, Weide GD, Tarasi M. Association between postoperative acute kidney injury and duration of cardiopulmonary bypass: a meta-analysis. J Cardiothorac Vasc Anesth. 2012;26(1):64–9. 10.1053/j.jvca.2011.07.007 . 21924633

[5] Arora P, Kolli H, Nainani N, Nader N, Lohr J. Preventable risk factors for acute kidney injury in patients undergoing cardiac surgery. J Cardiothorac Vasc Anesth. 2012;26(4):687–97. 10.1053/j.jvca.2012.03.001 . 22516466

[6] Perrotti A, Miltgen G, Chevet-Noel A, Durst C, Vernerey D, Bardonnet K, et al Neutrophil gelatinase-associated lipocalin as early predictor of acute kidney injury after cardiac surgery in adults with chronic kidney failure. Ann Thorac Surg. 2015;99(3):864–9. 10.1016/j.athoracsur.2014.10.011 . 25595830

[7] Perry TE, Muehlschlegel JD, Liu KY, Fox AA, Collard CD, Shernan SK, et al Plasma neutrophil gelatinase-associated lipocalin and acute postoperative kidney injury in adult cardiac surgical patients. Anesth Analg. 2010;110(6):1541–7. 10.1213/ANE.0b013e3181da938e 20435938 PMC2999841

[8] Lin X, Yuan J, Zhao Y, Zha Y. Urine interleukin-18 in prediction of acute kidney injury: a systemic review and meta-analysis. J Nephrol. 2015;28(1):7–16. 10.1007/s40620-014-0113-9 24899123 PMC4322238

[9] Kim K, Shin DG, Park MK, Baik SH, Kim TH, Kim S, et al Circulating cell-free DNA as a promising biomarker in patients with gastric cancer: diagnostic validity and significant reduction of cfDNA after surgical resection. Ann Surg Treat Res. 2014;86(3):136–42. 10.4174/astr.2014.86.3.136 24761422 PMC3994618

[10] Shoham Y, Krieger Y, Perry ZH, Shaked G, Bogdanov-Berezovsky A, Silberstein E, et al Admission cell free DNA as a prognostic factor in burns: quantification by use of a direct rapid fluorometric technique. Biomed Res Int. 2014;2014:306580 10.1155/2014/306580 25045663 PMC4090497

[11] Avriel A, Paryente Wiessman M, Almog Y, Perl Y, Novack V, Galante O, et al Admission cell free DNA levels predict 28-day mortality in patients with severe sepsis in intensive care. PLoS One. 2014;9(6):e100514 10.1371/journal.pone.0100514 24955978 PMC4067333

[12] Garnacho-Montero J, Huici-Moreno MJ, Gutierrez-Pizarraya A, Lopez I, Marquez-Vacaro JA, Macher H, et al Prognostic and diagnostic value of eosinopenia, C-reactive protein, procalcitonin, and circulating cell-free DNA in critically ill patients admitted with suspicion of sepsis. Crit Care. 2014;18(3):R116 10.1186/cc13908 24903083 PMC4229882

[13] Clark SR, Ma AC, Tavener SA, McDonald B, Goodarzi Z, Kelly MM, et al Platelet TLR4 activates neutrophil extracellular traps to ensnare bacteria in septic blood. Nat Med. 2007;13(4):463–9. 10.1038/nm1565 . 17384648

[14] Gupta AK, Joshi MB, Philippova M, Erne P, Hasler P, Hahn S, et al Activated endothelial cells induce neutrophil extracellular traps and are susceptible to NETosis-mediated cell death. FEBS Lett. 2010;584(14):3193–7. 10.1016/j.febslet.2010.06.006 . 20541553

[15] Brinkmann V, Reichard U, Goosmann C, Fauler B, Uhlemann Y, Weiss DS, et al Neutrophil extracellular traps kill bacteria. Science. 2004;303(5663):1532–5. 10.1126/science.1092385 . 15001782

[16] Saffarzadeh M, Juenemann C, Queisser MA, Lochnit G, Barreto G, Galuska SP, et al Neutrophil extracellular traps directly induce epithelial and endothelial cell death: a predominant role of histones. PLoS One. 2012;7(2):e32366 10.1371/journal.pone.0032366 22389696 PMC3289648

[17] van Montfoort ML, Stephan F, Lauw MN, Hutten BA, Van Mierlo GJ, Solati S, et al Circulating nucleosomes and neutrophil activation as risk factors for deep vein thrombosis. Arterioscler Thromb Vasc Biol. 2013;33(1):147–51. 10.1161/ATVBAHA.112.300498 . 23104849

[18] Kessenbrock K, Krumbholz M, Schonermarck U, Back W, Gross WL, Werb Z, et al Netting neutrophils in autoimmune small-vessel vasculitis. Nat Med. 2009;15(6):623–5. 10.1038/nm.1959 19448636 PMC2760083

[19] Margraf S, Logters T, Reipen J, Altrichter J, Scholz M, Windolf J. Neutrophil-derived circulating free DNA (cf-DNA/NETs): a potential prognostic marker for posttraumatic development of inflammatory second hit and sepsis. Shock. 2008;30(4):352–8. 10.1097/SHK.0b013e31816a6bb1 . 18317404

[20] Paunel-Gorgulu A, Wacker M, El Aita M, Hassan S, Schlachtenberger G, Deppe A, et al cfDNA correlates with endothelial damage after cardiac surgery with prolonged cardiopulmonary bypass and amplifies NETosis in an intracellular TLR9-independent manner. Sci Rep. 2017;7(1):17421 10.1038/s41598-017-17561-1 29234042 PMC5727170

[21] Likhvantsev VV, Landoni G, Grebenchikov OA, Skripkin YV, Zabelina TS, Zinovkina LA, et al Nuclear DNA as Predictor of Acute Kidney Injury in Patients Undergoing Coronary Artery Bypass Graft: A Pilot Study. J Cardiothorac Vasc Anesth. 2017;31(6):2080–5. 10.1053/j.jvca.2017.04.051 . 28967626

[22] Scrascia G, Guida P, Rotunno C, de Luca Tupputi Schinosa L, Paparella D. Anti-inflammatory strategies to reduce acute kidney injury in cardiac surgery patients: a meta-analysis of randomized controlled trials. Artif Organs. 2014;38(2):101–12. 10.1111/aor.12127 . 23876045

[23] Mehta RL, Kellum JA, Shah SV, Molitoris BA, Ronco C, Warnock DG, et al Acute Kidney Injury Network: report of an initiative to improve outcomes in acute kidney injury. Crit Care. 2007;11(2):R31 10.1186/cc5713 17331245 PMC2206446

[24] Grynberg K, Polkinghorne KR, Ford S, Stenning F, Lew TE, Barrett JA, et al Early serum creatinine accurately predicts acute kidney injury post cardiac surgery. BMC Nephrol. 2017;18(1):93 10.1186/s12882-017-0504-y 28302078 PMC5353965

[25] Ho J, Reslerova M, Gali B, Nickerson PW, Rush DN, Sood MM, et al Serum creatinine measurement immediately after cardiac surgery and prediction of acute kidney injury. Am J Kidney Dis. 2012;59(2):196–201. 10.1053/j.ajkd.2011.08.023 . 21967775

[26] De Loor J, Herck I, Francois K, Van Wesemael A, Nuytinck L, Meyer E, et al Diagnosis of cardiac surgery-associated acute kidney injury: differential roles of creatinine, chitinase 3-like protein 1 and neutrophil gelatinase-associated lipocalin: a prospective cohort study. Ann Intensive Care. 2017;7(1):24 10.1186/s13613-017-0251-z 28251598 PMC5332341

[27] Devarajan P. Biomarkers for the early detection of acute kidney injury. Curr Opin Pediatr. 2011;23(2):194–200. 10.1097/MOP.0b013e328343f4dd 21252674 PMC3257513

[28] Alge JL, Arthur JM. Biomarkers of AKI: a review of mechanistic relevance and potential therapeutic implications. Clin J Am Soc Nephrol. 2015;10(1):147–55. 10.2215/CJN.12191213 25092601 PMC4284423

[29] Parikh CR, Mishra J, Thiessen-Philbrook H, Dursun B, Ma Q, Kelly C, et al Urinary IL-18 is an early predictive biomarker of acute kidney injury after cardiac surgery. Kidney Int. 2006;70(1):199–203. 10.1038/sj.ki.5001527 . 16710348

[30] Qi Y, Uchida T, Yamamoto M, Yamamoto Y, Kido K, Ito H, et al Perioperative Elevation in Cell-Free DNA Levels in Patients Undergoing Cardiac Surgery: Possible Contribution of Neutrophil Extracellular Traps to Perioperative Renal Dysfunction. Anesthesiol Res Pract. 2016;2016:2794364 10.1155/2016/2794364 27882047 PMC5110877

[31] Gogenur M, Burcharth J, Gogenur I. The role of total cell-free DNA in predicting outcomes among trauma patients in the intensive care unit: a systematic review. Crit Care. 2017;21(1):14 10.1186/s13054-016-1578-9 28118843 PMC5260039

[32] Lindau D, Mussard J, Wagner BJ, Ribon M, Ronnefarth VM, Quettier M, et al Primary blood neutrophils express a functional cell surface Toll-like receptor 9. Eur J Immunol. 2013;43(8):2101–13. 10.1002/eji.201142143 . 23686399

[33] Zhang Q, Raoof M, Chen Y, Sumi Y, Sursal T, Junger W, et al Circulating mitochondrial DAMPs cause inflammatory responses to injury. Nature. 2010;464(7285):104–7. 10.1038/nature08780 20203610 PMC2843437

[34] Allam R, Scherbaum CR, Darisipudi MN, Mulay SR, Hagele H, Lichtnekert J, et al Histones from dying renal cells aggravate kidney injury via TLR2 and TLR4. J Am Soc Nephrol. 2012;23(8):1375–88. 10.1681/ASN.2011111077 22677551 PMC3402284

[35] Vaara ST, Lakkisto P, Immonen K, Tikkanen I, Ala-Kokko T, Pettila V, et al Urinary Biomarkers Indicative of Apoptosis and Acute Kidney Injury in the Critically Ill. PLoS One. 2016;11(2):e0149956 10.1371/journal.pone.0149956 26918334 PMC4769222

[36] Raup-Konsavage WM, Wang Y, Wang WW, Feliers D, Ruan H, Reeves WB. Neutrophil peptidyl arginine deiminase-4 has a pivotal role in ischemia/reperfusion-induced acute kidney injury. Kidney Int. 2018;93(2):365–74. 10.1016/j.kint.2017.08.014 29061334 PMC5794573

[37] Bennett M, Dent CL, Ma Q, Dastrala S, Grenier F, Workman R, et al Urine NGAL predicts severity of acute kidney injury after cardiac surgery: a prospective study. Clin J Am Soc Nephrol. 2008;3(3):665–73. 10.2215/CJN.04010907 18337554 PMC2386703

[38] Zappitelli M, Bernier PL, Saczkowski RS, Tchervenkov CI, Gottesman R, Dancea A, et al A small post-operative rise in serum creatinine predicts acute kidney injury in children undergoing cardiac surgery. Kidney Int. 2009;76(8):885–92. 10.1038/ki.2009.270 . 19641482

[39] Hoste EA, Blot SI, Lameire NH, Vanholder RC, De Bacquer D, Colardyn FA. Effect of nosocomial bloodstream infection on the outcome of critically ill patients with acute renal failure treated with renal replacement therapy. J Am Soc Nephrol. 2004;15(2):454–62. 10.1097/01.asn.0000110182.14608.0c . 14747393

[40] Parolari A, Camera M, Alamanni F, Naliato M, Polvani GL, Agrifoglio M, et al Systemic inflammation after on-pump and off-pump coronary bypass surgery: a one-month follow-up. Ann Thorac Surg. 2007;84(3):823–8. 10.1016/j.athoracsur.2007.04.048 . 17720383

[41] Dreischulte T, Morales DR, Bell S, Guthrie B. Combined use of nonsteroidal anti-inflammatory drugs with diuretics and/or renin-angiotensin system inhibitors in the community increases the risk of acute kidney injury. Kidney Int. 2015;88(2):396–403. 10.1038/ki.2015.101 . 25874600

[42] Lapi F, Azoulay L, Yin H, Nessim SJ, Suissa S. Concurrent use of diuretics, angiotensin converting enzyme inhibitors, and angiotensin receptor blockers with non-steroidal anti-inflammatory drugs and risk of acute kidney injury: nested case-control study. BMJ. 2013;346:e8525 10.1136/bmj.e8525 23299844 PMC3541472

[43] Martensson J, Bellomo R. The rise and fall of NGAL in acute kidney injury. Blood Purif. 2014;37(4):304–10. 10.1159/000364937 . 25170751

[44] Andreev E, Koopman M, Arisz L. A rise in plasma creatinine that is not a sign of renal failure: which drugs can be responsible? J Intern Med. 1999;246(3):247–52. . 10475992 10.1046/j.1365-2796.1999.00515.x

[45] Ferreiro A, Lombardi R. Acute kidney injury after cardiac surgery is associated with mid-term but not long-term mortality: A cohort-based study. PLoS One. 2017;12(7):e0181158 10.1371/journal.pone.0181158 28700753 PMC5507329

[46] Petaja L, Vaara S, Liuhanen S, Suojaranta-Ylinen R, Mildh L, Nisula S, et al Acute Kidney Injury After Cardiac Surgery by Complete KDIGO Criteria Predicts Increased Mortality. J Cardiothorac Vasc Anesth. 2017;31(3):827–36. 10.1053/j.jvca.2016.08.026 . 27856153

[47] Moriyama T, Hagihara S, Shiramomo T, Nagaoka M, Iwakawa S, Kanmura Y. Comparison of three early biomarkers for acute kidney injury after cardiac surgery under cardiopulmonary bypass. J Intensive Care. 2016;4:41 10.1186/s40560-016-0164-1 27330813 PMC4915135

